# AUGMENTATION INDEX IS A PREDICTOR OF CEREBRAL BLOOD FLOW ACROSS GLOBAL GRAY MATTER IN THE ELDERLY

**DOI:** 10.1093/geroni/igz038.2390

**Published:** 2019-11-08

**Authors:** Adrian Noriega de la Colina, Badji Atef, Sven Joubert, Louis Bherer, Maxime Lamarre-Cliche, Claudine Gauthier, Julien Cohen-Adad, Hélène Girouard

**Affiliations:** 1 Research Centre of the IUGM, University of Montreal, Montréal, Québec, Canada; 2 NeuroPoly Lab, Institute of Biomedical Engineering, Polytechnique Montreal, Montréal, Québec, Canada; 3 Centre de recherche de l’Institut Universitaire de Gériatrie de Montréal, Montréal, Québec, Canada; 4 Montreal Heart Institute, Montreal, Quebec, Canada; 5 Institut de Recherches Cliniques de Montréal, Université de Montréal, Montreal, Quebec, Canada; 6 PERFORM Centre, Concordia University, Montreal, Quebec, Canada; 8 Department of Pharmacology and Physiology, Faculty of Medicine, Université de Montréal, Montréal, Québec, Canada

## Abstract

Arterial stiffness and blood pressure (BP) are contributors to cognitive decline and dementia. Lower global cerebral blood flow (CBF) is one of the earliest manifestations of biological alterations linked to cognitive decline, nevertheless the best cardiovascular predictor of CBF in gray-matter (CBF-GM) remains to be identified. Our objective is to determine the best predictor of CBF-GM levels amongst cardiovascular parameters. Eigthy-four healthy participants between 60-80 years-old were evaluated. The measured parameters for arterial stiffness were the carotid-femoral pulse wave velocity (cf-PWV) and the augmentation index (Aix), measured by applanation tonometry (SphygmoCor). Mean systolic BP (SBP) was monitored over 24-hours and analyzed following Hypertension-Canada’s guidelines(2018). The coefficient of variation for 24-hours SBP was calculated by dividing the standard deviation by the mean. Resting CBF-GM was quantified from pseudocontinuous arterial-spin-labeling using Neurolens 2.0(pcASL), and acquired on a 3T scanner (MAGNETOM Prisma-Fit). We created multiple linear regression models for each independent variable (cf-PWV, Aix, mean-SBP in 24 hours and the coefficient of variation of 24-hours SBP) using age, sex, schooling and body mass index as covariates. Multiple linear regression models demonstrated that two independent variables could predict CBF-GM levels: a)PWV (p=0.010) and b)Aix (p=0.006). In this cohort, we demonstrated that while PWV and Aix are both predictors of CBF-GM levels, it is Aix which has the highest predictive value and could be a useful tool to understand the interplay between lower CBF-GM and arterial stiffness. These results also indicate that Aix may be a good therapeutic target to preserve brain health and cognition.

